# Effect of negative emotions in consumption during the COVID-19 pandemic: A study from Peru

**DOI:** 10.1371/journal.pone.0293932

**Published:** 2023-11-03

**Authors:** Otto Regalado-Pezúa, Orly Carvache-Franco, Mauricio Carvache-Franco, Wilmer Carvache-Franco, Maribel Ortiz-Soto, Guisell Larregui-Candelaria

**Affiliations:** 1 ESAN Graduate School of Business, Universidad ESAN, Lima, Peru; 2 Facultad de Economía y Empresa, Universidad Católica de Santiago de Guayaquil, Guayaquil, Ecuador; 3 Universidad Espíritu Santo, Samborondón, Ecuador; 4 Facultad de Ciencias Sociales y Humanísticas, Escuela Superior Politécnica del Litoral, ESPOL, Guayaquil, Ecuador; 5 Ana G. Mendez University, San Juan, Puerto Rico; 6 Instituto Tecnológico de Puerto Rico, Manatí, Puerto Rico; University of Haifa, ISRAEL

## Abstract

The research examines the negative consumer emotions generated by the perception of social networks or traditional media with consumer behavior during the covid_19 pandemic. The study was developed in Peru with a sample of 220 consumers; the design is quantitative and structural equations were used for data processing. The results indicate that social networks and traditional media are not related to negative emotions, but are related to the change in consumer behavior in the purchase of more products and new products. The research has theoretical implications since it provides evidence to the literature that the negative emotions generated during the covid_19 pandemic are related to changes in consumer behavior, which affect the purchase of more products and new products. The practical implications of the research is for businessmen on the causes of changes in consumer behavior generated during crises. like the COVID-19 pandemic.

## 1. Introduction

The context of the covid_19 pandemic generated negative emotions such as unhappiness, sadness, shame, fear and anger that have developed influenced by social and traditional media. In crisis conditions such as during pandemics, consumers adopt changes in their behavior to protect themselves from crises and negative emotions such as fear, anger, anguish and anxiety arise.+

Consumers receive information from family and friends about covid_19 through different communication channels, such as social networks and traditional media. This information generates negative emotions, which drive consumers to change their purchasing behavior in different categories.

Panic buying due to the pandemic has increased the demand for products in several nations and economies during the covid_19 pandemic [1], and its effects have been studied from various dimensions: such as the effect of fear and dread [1] environmental stimuli such as reflective thinking [2], uncertainty, severity and scarcity [3], threat of crisis and scarcity, fear of the unknown and social psychological factors [4].

Negative emotions such as fear, restlessness and uncertainty created panic buying during the covid_19 pandemic, which are purchases of larger quantities of products and new products. There is a gap in the literature on whether the negative emotions generated in consumers by social networks or traditional media affect or modify the purchasing behavior of new products and products in greater quantity during the pandemic.

The objective of the research is to find out if these negative emotions generated during the pandemic are related to changes in consumer behavior that produce changes in the purchase of products in quantity and new products. This research is carried out in Peru, an emerging economy, to contribute to the literature gap since studies in this type of countries are scarce in developing countries since consumers can adopt different behaviors from consumers in developed countries.

## 2. Literature review

People tend to feel nervous and insecure when experiencing especially environmental changes [5]. In the case of infectious disease outbreaks, when the causes of disease progression and outcomes are uncertain, they give room for rumor spreading and closed-minded attitudes [6]. These responses lead to fear and uncertainty, bringing negative social feelings and behaviors as consequences [7]. Fear, anxiety, or perceived scarcity may prompt panic buying or hoarding as mitigation mechanisms for perceived risk and negative emotions aroused by the prevailing situation [8, 9].

Negative feelings can alter consumer behavior as consumption is a habit, and social context can disrupt consumer habits [9]. Likewise, sociologists and psychologists have researched and documented that life transition periods are critical phases in a person’s life and are associated with significant behavioral changes [10, 11].

Recent significant developments and trends [12] argue that the consumer landscape operates, in the new times, in a rapidly changing environment and can be described as turbulent and disruptive. Likewise, in these scenarios, significant events are taking place that alter how consumers behave. It is surprising to realize how much the consumer landscape can change from the routine. Stressful events result in the initiation, intensification, or changes in consumption habits to manage the stress caused by social change [13].

One of the changes that can be observed is panic buying, which occurs when consumers purchase vast quantities of products in anticipation of, during, or after a disaster or in anticipation of an increase or decrease in the prices of needed products [4]. Panic alludes to intense collective fear and connotes primitive, disorderly, and even violent actions in a catastrophe [14].

Panic buying is a socially undesirable behavior in which large quantities of necessities and medical supplies are purchased in markets, often giving rise to situations of shortages [15]. Panic buying by consumers has the potential to exaggerate the consequences of supply disruption [16]. They increase consumer anxiety about supply shortages and worsen panic buying [17]. Sheu & Kuo [18] pointed out that panic buying, or other behaviors, is more about mass behaviors than mixing rational aspects with irrational and emotional ones.

Regarding stockpiling more than current consumption needs, Sheth [9] noted that consumers have two motives for stockpiling items: (i) as stock to protect against stock-outs given uncertainty about future usage needs, and (ii) for economic reasons, i.e., the convenience of stocking up on storable goods when a supply becomes available, i.e., when retailers offer goods at a relatively low price.

The covid_19 pandemic increased depression in people, and in this state receiving negative messages from news or advertisements caused a greater negative impact on people, causing them greater anxiety and panic or fear, which alters their consumption behavior [19]. The main changes in consumer behavior during the pandemic were: abnormal purchasing behavior, changes in product preferences, greater use of technology and digital media to make purchases [20]. Various intentions of consumption behaviors have emerged with covid_19 influenced by fear and hope, such as behavior focused on health, conscious consumption and support for local products, this mainly due to the vulnerability they perceived, which is why they acted with actions of protection. protection [21]. Covid_19 caused an impact on the change in the lifestyle and purchasing behavior of consumers influenced by the socioeconomic environment of consumers and has been proven to have a greater impact on consumers from less organized sectors, which caused an increase in substitute products for daily activities [22]. Consumer purchasing behavior mainly impacts sustainable products. During the pandemic, there was greater awareness, concern and environmental habits, so consumers were predisposed to pay more for sustainable products, but the change is affected by the demographic variables such as gender, age, income level and education [23].

The theory of panic-created behavior by Schultz [24], was compared with other theories put forward by colleagues/experts of the time [25]. Scholars have widely mentioned and studied this behavior in the wake of the covid_19 pandemic [26–29]. Addo et al. [30] further noted that panic buying is expected to lead to price changes in times of crisis, such as the current one caused by covid_19.

Furthermore, it is imperative to understand the impact and course of the pandemic caused by covid_19 on panic buying [30]. There are psychological and economic explanations for this stockpiling behavior in a crisis. A common psychological explanation is that accumulating storable goods gives consumers a sense of control over the risky situation created by a crisis [31].

Panic buying represents a relatively unexplored area in consumer behavior research, where purchase decisions are affected by emotions, such as fear of the unknown, anxiety, and social influences [32, 33]. During the covid_19 pandemic, panic and fear due to the excessive increase in prices of various products and the fear of greater shortages caused social influence to change consumer behavior to increase their purchases of some products [34].

Cohen [35] substantiates the theory of moral panic based on the reaction of a group of people to a perception that creates fear. A moral panic occurs when a condition, episode, person, or group of people emerges to be defined as a threat to social values and interests [31]. In extreme cases, moral panic creates mass hysteria within society, and the general public begins to believe that everything reported is happening everywhere [31].

Cohen [10] established five stages of moral panic: (1) something or someone is defined as a threat to values or interests, (2) this threat is represented in an easily recognizable form by the media, (3) there is a rapid build-up of public concern, (4) there is a response from authorities or opinion makers, and finally (5) the panic recedes or results in social change.

### 2.1. Effect of culture on consumer purchasing behavior

According to Sanz Blas et al. [36], there are factors involved in adopting online shopping innovation; one of them is culture, as it represents a set of shared values that can influence consumer perceptions, attitudes, preferences, and responses. Consumers can be affected by high or low-context cultures and collectivist or individualistic cultures [37].

The Latin American consumer differs in many ways from consumers in other parts of the world. One of the characteristics is the attachment or bond they have with the things or objects they acquire; this is because they are conservative, and it is difficult for them to get rid of an object even if it has gone out of fashion or is obsolete. They consider their belongings extensions of themselves, and affective bonds are generated towards what they acquire [38].

The main concern of Latin American consumers is economic uncertainty. Over and above the covid_19 health crisis, many consumer attitudes have changed, and five behavioral changes have been observed: the first is Mindful Consumption, which refers to the consumer being more attentive to the value of the products they consume; the second is Always Mobile which refers to the new consumer making more purchases on digital platforms; the third is Eco Doing, which is based on the concern for environmental and social sustainability; and the fourth is Responsumers, which reflects that people are more demanding with brands, companies, institutions, the fifth is Wellbeing Reloaded, which refers to new habits of integral wellbeing, such as concern for the food’s origin [39].

### 2.2. Consumer behavior of Peruvian shoppers

Bardales and Herrera [40] considered that the Peruvian consumer had become a net prosumer, he identifies with brands, but now he wants brands to identify with him. It can even destroy a brand, as it did with the case of Domino’s Pizza^®^, except love brands unfaithful by knowledge and increasingly more rational when choosing offers and comparing with the information they have at hand. Peruvian consumers are more demanding when buying and have more power than before, especially in social networks. They have the information and a more remarkable ability to demand and therefore believe that a future trend will be the increase of this demand.

According to Alvarez [41], confinement and social distancing interrupted interpersonal relationships and missed family and friends meetings. Outdoor activities are increasingly revalued; returning to shopping malls and restaurants with the confidence and security of the case would be highly appreciated. Most Peruvians state that a vaccine passport should be required to enter them, even in large spaces such as stadiums or in small ones such as stores and offices.

Navarro [42] points out that during the crisis, many consumers changed several of their habits: trying new brands/products instead of the ones they used to buy, and these changes may make them try other products, evaluate the price and performance to probably consider them in the future within their usual shopping list. What will be then their new shopping habits in the future?

### 2.3. Communication channels (traditional social networks) and their influence on Peruvian consumers’ purchasing behavior

For Okazaki et al. [43], the emergence of social networks has significantly impacted how companies promote their products and services and consumers’ decision-making process regarding their purchases—using the application and extension of the proposed models. Consumer behavior during the covid_19 pandemic was affected by various environmental stimuli, such as social networks that, together with other stimuli such as the economic recession, partial lockdown regulations and restrictions on some services, influenced a change in behavior in the purchase of consumer goods, less impulsive and more planned companies, less frequent purchases [44].

According to Pfeiffer & Zinnbauer [45], recipients also have communication channel preferences. It is difficult for advertisers to measure the effectiveness and results of marketing campaigns, especially when using traditional communication channels in the service sector, because it creates a challenge for the marketing decision-maker to allocate the marketing budget most efficiently.

### 2.4. Effect of the covid_19 pandemic crisis on the Peruvian consumer’s purchase behavior (new products, quantity of products)

Nielsen [46] explained that consumer habits in Latin America were mainly marked by socioeconomic factors affected by rising unemployment and change in the economy. They identified five predictive factors in the purchase process: readjustment of the basket, increased purchase of digital formats, increased consumption at home, more empathetic brands, and search for reactivation. Similarly, [47] noted that, concerning consumption habits, the arrival of covid_19 brought changes in purchasing behavior, including a 29% increase in spending on food, 15% on dairy products, and 12% on home care items. The most consumed food is flour.

The behavior of Peruvian consumers during covid_2019 was different, since they tried to avoid waste, when making purchases they were oriented on cost-benefits, carrying out prior purchasing planning with knowledge of the labels and storage of products, they also considered their own culinary skills for these products [48]. The covid_19 pandemic shows the resilience to everyday consumption rooted in the family to the extent that new rules and norms were imposed in society, so new consumption habits were acquired by consumers [49].

According to Kantar [39] on household consumption habits, the most critical finding was the comparison of purchased tickets in comparison of the years 2019, 2020, and 2021. The first comparison of the first quarter between the years 2019 and 2021 showed a positive transformation; however, in the same comparison of tickets for the same period (1st quarter) of the years 2020 and 2021, the growth was higher (25%) even achieved before the pandemic.

In a previous study in the Peruvian market during the covid_19 pandemic, it was found that there was a change in consumer purchasing behavior influenced by social factors, that is, by external influences on society, and by psychological factors in the population, while no incidence was found. of cultural factors and personal factors in purchasing behavior [50]. In other Latin American countries, it has been found that the covid_19 pandemic had an impact on the flow of sustainable consumption, the consumer purchases with greater environmental awareness and social responsibility [51].

### 2.5 Study hypothesis

Because consumers’ prolonged exposure to adverse reports on social media during crises can impact fears and negative emotions such as fear and panic [52], social media can alter consumers’ emotions, as risky situations are perceived during crises [53], the following hypothesis is proposed.

H1 = Social media positively affects negative emotions.

Traditional media during crises contribute to fear and panic and produce emotions in consumers influenced by panic [54], producing emotions and influencing the audience [55]. The following hypothesis is proposed.

H2 = Traditional media positively impact negative emotions.

Considering that fear, anxiety, or perceived scarcity can propitiate change in consumer behavior. Emotions, such as panic buying or hoarding, act as mitigation mechanisms of perceived risk and prevailing situations [8, 9], so negative emotions can alter consumer behavior [18]. The following hypothesis is proposed.

H3 = Negative emotions impact changes in consumer behavior.

It is considered that negative emotions produced by fear produce panic purchases that generally correspond to the acquisition of more products to mitigate the risk and situation [8, 9]. The following hypothesis is proposed.

H4 = Changes in consumer behavior impact the purchase of new products.

It is considered that negative emotions produced by fear produce panic purchases that generally correspond to the accumulation of products to mitigate the risk and situation [8, 9]. The following hypothesis is proposed.

H5 = Changes in consumer behavior impact the purchase of more products.

**Fig 1:**
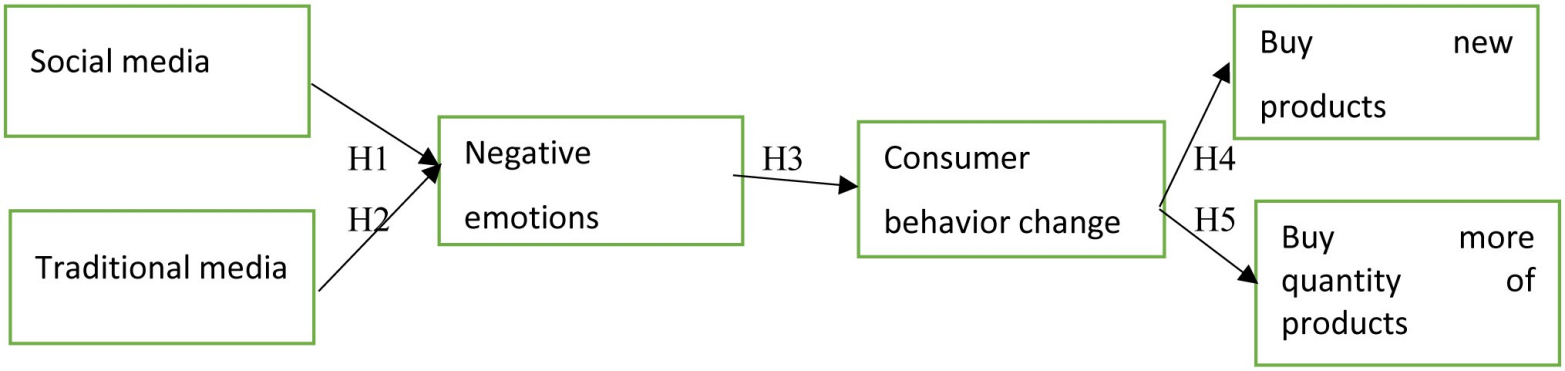
Conceptual model.

Fig 1 below shows the conceptual model for observing the variables and hypotheses.

## 3. Methodology

In order to evaluate the various relationships of the hypotheses, the following conceptual model of Fig 1 is proposed, which shows the various relationships mentioned in the hypotheses.

### 3.1. Design and instrument

The design used is quantitative, non-experimental, and cross-sectional. The questionnaire is composed of the following parts (1) demographic part with questions on gender, age range, degree of schooling, education level, and income range (2) communications through social media (3) communications through traditional media, (4) negative emotions (5) changes in consumer behavior (6) new products and (7) more quantity of products. The scale used is the 5-point Likert scale. The evaluation instrument was developed by the researchers after analyzing the literature review looking for the statements included to answer the objectives of the study. After the instrument was designed, it was validated by 5 experts in marketing and research methodology. These experts evaluated the validity of the instrument, ensuring comprehension, proper order of the questions, and that the instrument measured the proposed objectives. The recommendations given by this group were incorporated and reviewed in the instrument.

The validity and reliability of the questionnaire was evaluated with a representative group of the population using a pilot test with 40 people. With the results obtained from the test, a PLS was carried out to preliminarily evaluate the behavior of the variables and the model. Using the results of the PLS, the reliability and initial validity of the instrument were calculated. Firstly, the loads obtained (Factor Loading) were analyzed. The results of the loads are obtained from the calculation of the SmartPLS algorithm, all the results less than 0.40 are eliminated to comply with the rule of Hair et al. [56], and the process was repeated, computing the SmartPLS algorithm again, obtaining results where all the charges were above 0.40. Then the Alpha coefficients and the convergent validity of each variable were analyzed and the results reflected that the majority met the .70 criterion as established by Hair et al. [56] and Henseler et al. [57]. The behavior change variable obtained a Cronbach’s Alpha of 0.63. However, Hair et al. [58] clarify that a value between 0.60 and 0.69 indicates that the value, although it is a weak one, may be acceptable; a value below 0.59 is considered an unacceptable value to carry out the investigation. In the same way, the AVE values mostly reflected results above 0.50, concluding that the latent variables explained more than half of the variance on their indicators, according to the 0.50 criterion of Hair et al. [56], with the exception of the variables products with more quantity (0.45) and new products (0.47). However, Hair et al. [58], clarifies that the values can be between 0.40 to 0.70.

### 3.2. Data collection

The questionnaire was also ethically approved by the ESAN Graduate School of Business of Peru and included the participants’ written informed consent. The sample comprised 220 people and was taken in Peru in 2021. The sampling used was non-probabilistic and of convenience. The questionnaire was administered online via SurveyMonkey.

The population object of the investigation will be men and women over 21 years of age residing in Peru. To determine the research sample, "10 times the rule" was used [59], which indicates that the sample size must be equal to the greater than 10 times the largest number of formative indicators used to measure a single construct or 10 times the largest number of structural pathways in a particular construct targeted in the structural model [58]. Therefore, it is equivalent to saying that the minimum sample size should be 10 observations for each relationship. On this rule, it was determined that the sample size will be 200 duly completed questionnaires for the investigation. The researchers have established a 95% confidence level and a 5% confidence interval. Among the inclusion criteria for this research must be men or women over 21 years of age. In the exclusion criteria is that the participants are under 21 years of age.

### 3.3. Data processing

A confirmatory factor analysis is applied, and criteria of reliability, convergent and discriminant validity are considered. Reliability is assessed based on composite reliability (CR), that is, the degree to which items are free of error and therefore produce reliable results, using CR > = 0.70 as the appropriate value [60]. Convergent validity is verified using factor loadings greater than 0.5 and a minimum variance (AVE) of 0.5, while discriminant validity is determined by a minimum value of AVE = 0.5 [60].

Subsequently, the method of structural equations is applied to evaluate and test the relationships proposed in the hypotheses and to evaluate the model, indices are selected as Comparative Fit Index CFI, Goodness of Fit Index (GFI), and Normed Fit Index (NFI), used as a measure of comparison that CFI > = 0.9 [61], GFI > = 0.9 [62] and NFI > = 0.9 [61]. Likewise, the root mean square error of approximation (RMSEA) index was used, considering an appropriate measure of 0.05 to 0.08 [62–64].

The AMOS software is used to perform path analysis or the analysis of the relationships between variables, determining the coefficient (β) and the standard error (S.E) and the p-value. To accept the hypotheses, those with a p-value or significance less than 0.05 are considered supported or accepted.

## 4. Results

Descriptive results were determined and are shown in Table 1, in which it is determined that in the sample, broken down by gender, men represent 55.90%. The age group that predominates in the sample is 41–55 years old, with 38.6%. Regarding schooling level, those with an associate or technical degree predominate with 34.5%, and those with a high school degree with 42.3%. Regarding annual income level, most earn between USD20,001 and USD35,000 (25.50%).

**Table 1:** Descriptive data.

Socio-demographic variable	Frequency	Percent (%)
Gender	220	100
Male	123	55.90
Female	97	44.10
Age (years)	200	100
21 a 40	114	51.8
41 a 55	85	38.6
56 a 74	19	8.6
75 or more	2	0.9
Grade of schooling	220	100
Doctorate	0	0
Master’s degree	21	9.5
Bachelor’s degree	22	10
Associate degree or technical course	76	34.5
High school	93	42.3
Less than high school education not completed	8	3.6
Estimated annual income	220	100
Less than USD10,000	39	17.70
USD10,001 a USD20,000	47	21.40
USD20,001 a USD35,000	56	25.50
USD35,001 a USD50,000	32	14.50
USD50,001 a USD65,000	21	9.50
USD65,001 or more	25	11.40

In relation to the confirmatory factorial analysis, Table 2 shows the CR values obtained between 0.75 and 0.94, which are acceptable values for CR > 0.70, AVE value and values of factor loads that are greater than 0, 50, which meets the criterion of good convergent validity and verifies that the values of the explained mean variance are more significant than 0.5, which indicates good convergent validity.

**Table 2:** Reliability and factor loadings.

	Constructs /Measurement Items	Standardized factor Loadings	CR	AVE
Social media			0.84	0.76
RS1	I pay more attention to information about covid_19 received through social networks sent by my friends and family.	0.73		
RS2	The information received through social networks from friends and family is more accurate.	0.92		
RS3	The information received through social networks by friends and family about covid_19 is more important than other media.	0.79		
RS4	The information about covid_19 received through social networks is the most important.	0.60		
Traditional media			0.94	0.86
CT1	I pay more attention to information about covid_19 received through phone or cell phone calls sent by my friends and family.	0.78		
CT2	The information received through phone or cell phone calls from friends and family is more accurate.	0.82		
CT3	The information received through phone or cell phone calls from friends and family about covid_19 is more important than other media.	0.85		
CT5	I pay more attention to information about covid_19 received through text messages from my friends and family.	0.92		
CT6	Information received via text messages from friends and family is more accurate.	0.91		
CT7	Information received via text messages from friends and family about covid_19 is more important than other media.	0.88		
Negative emotions			0.776	0.78
EN1	The information related to covid_19 received through the different media irritates me.	0.78		
EN8	The information about covid_19 received through the different media makes me anxious.	0.82		
EN15	The information related to covid_19 received through the different media frightens me.	0.85		
EN16	The information about covid_19 received through the different media makes me feel scared.	0.92		
EN17	The information about covid_19 received through the different media makes me fearful.	0.91		
EN18	The information about covid_19 received through the different media makes me feel scared.	0.88		
Consumer behavior change			0.54	0.41
CO1	I have changed my shopping habits.	0.43		
CO2	I organize products in order of importance before I buy them (I didn’t do it before)	0,53		
CO3	I now buy more products than before	0.34		
CO5	I make a list to buy what I need (I didn’t before).	0,33		
New products			0.834	0,70
NP1	Due to the feeling generated by the information related to covid_19, I have decided to buy new products in the following categories: basic food basket.	0.58		
NP3	Because of the sentiment generated by the information related to covid_19, I have decided to buy new products in the following categories: personal care and hygiene.	0.84		
NP4	Based on the sentiment generated by the information related to covid_19, I have decided to buy new products in the following categories: household cleaning.	0.60		
NP7	Because of the sentiment generated by the information related to covid_19, I have decided to buy new products in the following categories: medicine.	0.90		
NP8	Because of the sentiment generated by the information related to covid_19, I have decided to buy new products in the following categories: body protection against diseases.	0.60		
More quantity of products			0.828	0.74
MP1	Due to the feeling generated by the information related to covid_19, I have decided to buy more products than usual in the following categories: basic shopping cart.	0.51		
MP3	Because of the sentiment generated by the information related to covid_19, I have decided to buy more products than usual in the following categories: personal care and hygiene.	0.89		
MP4	Because of the sentiment generated by the information related to covid_19, I have decided to buy more products than usual in the following categories: household cleaning.	0.93		
MP8	Because of the sentiment generated by the information related to covid_19, I have decided to buy more products than usual in the following categories: body protection against diseases.	0.63		

The fit of the model was verified. The χ2/df ratio was calculated to be 3.01, considered adequate since an acceptable value of χ2/df < = 3 [53]. The CFI, GFI, and NFI indices were checked and obtained values of CFI = 0.822, close to the comparison value of 0.9 [50], GFI = 0.82, close to the comparison value of 0.9 [51], and NFI = 0.756 close to the comparison value 0.9 [50] so they are considered acceptable values and the RMSEA value was 0.09 also a value close to the reference value of 0.08 [51–53]. The model is considered to have a good fit level with these values obtained. Table 3 shows the values obtained from the model fit.

**Table 3:** Results of the fitting model.

Fit Indices	Benchmark	Value
**Absolute fit measures**		
CMIN (χ 2)		**1125.10**
DF		**373**
CMIN (χ 2)/DF	**3**	**3.01**
GFI (Goodness of Fit Index)	**0.9**	**0.82**
RMSEA (Root Mean Square Error of Approximation)	**0.08**	**0.09**
**Incremental fit measures**		
AGFI (Adjusted Goodness of Fit Index)	**0.80**	**0.697**
NFI (Normed Fit Index)	**0.90**	**0.756**
CFI (Comparative Fit Index)	**0.90**	**0.822**
IFI (Incremental Fit Index)	**0.90**	**0.823**
RFI (Relative Fit Index)	**0.90**	**0.735**
**Parsimonious fit measures**		
PCFI (Parsimonious Comparative of Fit Index)	**0.50**	**0.755**
PNFI (Parsimonious Normed Fit Index)	**0.50**	**0.695**

Each hypothesis was evaluated by obtaining the coefficient β and the p-value or probability value for each relationship. Table 4 shows the result of the path analysis. It is shown that the negative path emotions and social media, with a coefficient β = 0.054 and a p-value of 0.489 greater than 0.05, does not support hypothesis H1. While the negative path emotions and traditional media, with a coefficient β = -0.008 and a p-value of 0.915 greater than 0.05, supports hypothesis H2.

**Table 4:** Results of hypothesis testing.

Path	β	C.E.	C.R.	P	Result
(H1) Negative emotions <--- Social networks	0.054	0.077	0.699	0.489	Not Supported
(H2) Negative emotions <--- Traditional media	-0.008	0,076	-0.107	0.915	Not Supported
(H3) Consumer behavior change <--- Negative emotions	0.126	0.034	3.712	***	Supported
(H4) New products<--- Consumer behavior change	1.505	0.314	4.786	***	Supported
(H5) More quantity of products<-- Consumer behavior change	1.668	0.331	5.040	***	Supported

These results show that hypotheses H3, H4, and H5 are supported by the model.

H3 = Negative emotions affect changes in consumer behavior.

H4 = Changes in consumer behavior affect the purchase of new products.

H5 = Changes in consumer behavior affect to the purchase of more products.

**Fig 2:**
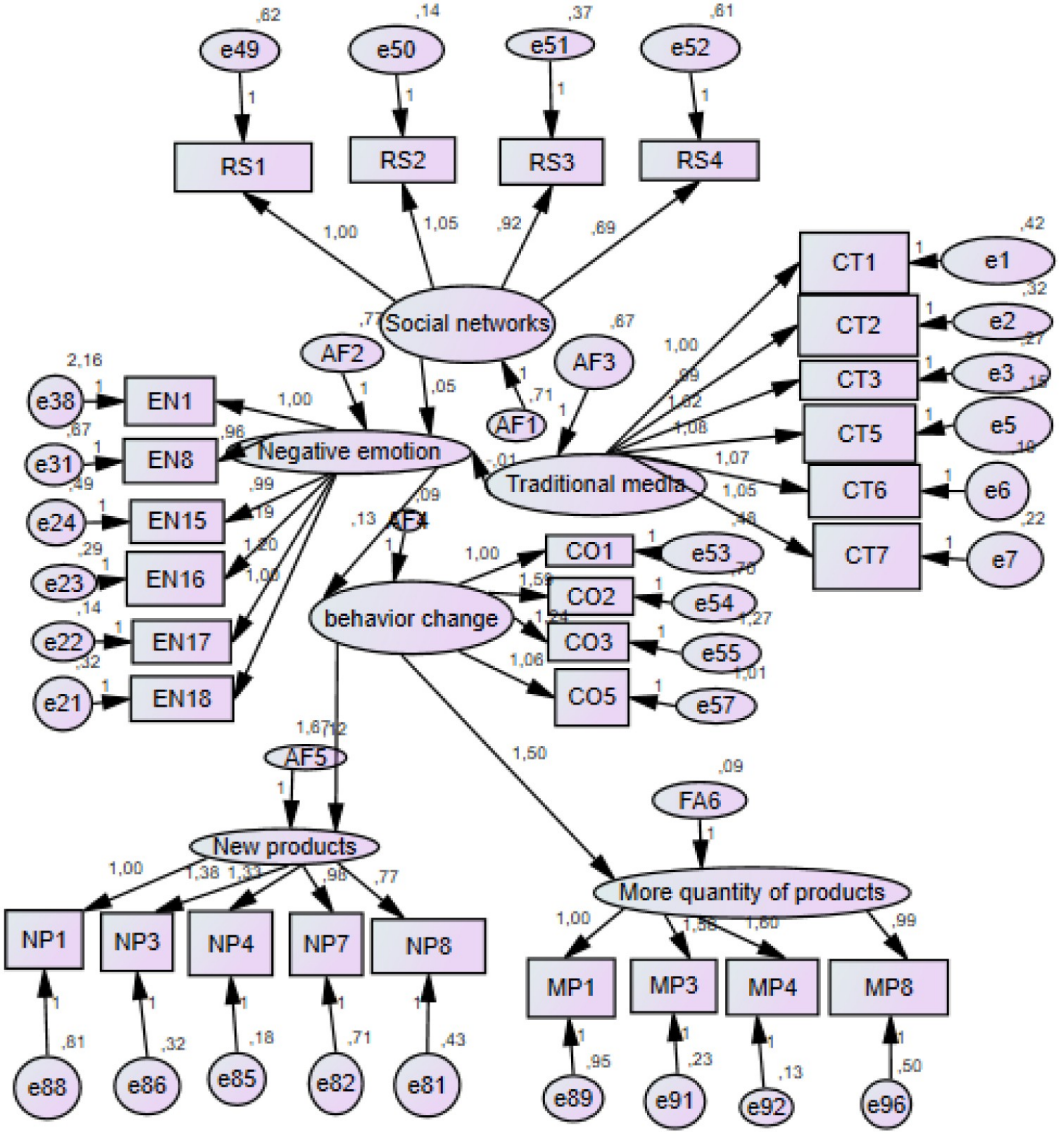
Structural equation model.

In contrast, the path changes in consumer behavior and negative emotions, with a coefficient β = 0.126 and a p-value less than 0.05; therefore, hypothesis H3 is supported. The path of new products and changes in consumer behavior, with a coefficient β = 1.505 and a p-value less than 0.05, shows that hypothesis H4 is supported. Finally, the path more products and changes in consumer behavior, with a coefficient β = 1.668 and a p-value less than 0.05, supports hypothesis H5. The structural model is shown in Fig 2.

## 5. Discussion

The research aims to examine the relationship between social media and traditional media on consumers’ negative emotions during the covid_19 pandemic and whether consumers’ negative emotions are related to the change in consumer behavior that affects the purchase of products in terms of quantity and new products.

The results of hypotheses H1 and H2 indicate that social media and traditional media are not related to consumers’ negative emotions during the covid_19 pandemic; this is justified because other causes could have contributed to generating negative emotions during the pandemic, such as environmental stimuli and reflective thinking [2], perception of uncertainty, severity, and scarcity [3], crisis threat and scarcity, fear of the unknown, social psychological factors [4] generated in the social environment that not necessarily that originate from social media or traditional media.

The results of hypotheses H3, H4, and H5 indicate that the negative emotions generated by consumers during the pandemic covid_19 changed consumer behavior that affected the purchase of new products and more products. These results agree with Sheth [9], who mentioned that fear and dread as negative emotions generated during the covid_19 pandemic caused changes in consumer buying behavior as they resorted to impulse purchases and are in agreement with [4], who mentioned that there are psychological causes that produce panic in consumers during crises. These change their purchasing behavior in acquiring more significant quantities of products because of the crisis and agree with [65], who mentioned that consumers, as a form of defense, buy larger quantities of products than usual during crises. Similarly, they also buy new products because they perceive that products may be in short supply.

The changes in consumer behavior are explained by George and Dane [66], who indicated that emotions in consumers produce changes in their buying behavior and [67] that emotions can vary rationality in people. Additionally, Willman-Iivarinen [68] mentions that consumer buying choice is affected by factors such as time pressure and buying opportunities, both identified in crises such as covid_19.

According to Amalia et al. [69], people are different, as is their perception of a situation, and risk perception reflects the buyer’s interpretation of their consumption. As mentioned by Bagozzi et al. [70], the messages and perceptions that consumers receive produce negative and positive emotions before purchases, which are mixed and form the anticipated emotions that impact the consumer’s purchase decision.

This research contributes to the literature because although it is known that the covid_19 pandemic generated panic purchases produced by fear, panic, and negative emotions [1], little is known if social media and traditional media related to negative emotions in consumers during the covid_19 pandemic, and if these negative emotions generated in the context of the covid_19 pandemic are related to higher quantity purchases and purchases of new products, in other economies.

## 6. Conclusions

Fear is a great motivator that, depending on its effect, can cause very specific changes in consumer buying behavior. The interpretation of fear can be a very particular purchase motivator. Therefore, the emotion of fear must be understood from its different dimensions. Each crisis is different and the peculiarity of each one of them is what can define the new purchasing behavior of the consumer.

The COVID 19 pandemic generated great fear in the consumer, inducing a change in behavior in terms of increasing the amount of product and purchasing new products. The results show that each crisis can prompt the consumer to change their purchasing behavior according to their interests and concerns.

The research concludes that in Peru social networks and traditional media are not related to the negative emotions of consumers during the covid_19 pandemic, that is, these media did not have a strong influence on the perception of risk or the generation of fear due to the crisis of the pandemic. In addition, the research finds that the negative emotions that consumers had during the covid_19 pandemic are related to changes in consumer behavior and have a positive effect on the purchase of more products and purchases of new products.

The research has theoretical implications because it contributes evidence in the context of Peru. The effect of social media and traditional media, such as newspapers and radio, on negative emotions during the covid_19 pandemic contributes to the evidence that the negative emotions of consumers during the covid_19 pandemic affect purchases of a higher quantity of products and new products.

This research has practical implications for business managers and academics, as they can learn about the changes in consumer behavior resulting from negative emotions that consumers may have and how this affects product quantity purchases and new product purchases, which can be helpful for sales planning during crises such as the covid_19 pandemic.

The study contributes from the perspective that store owners and marketing specialists must understand the type of emotion that consumers feel in a crisis such as the covid_19 pandemic so that they can design strategies that meet their expectations and the needs of companies and of the clients.

This research has limitations due to the temporality of the data that was taken during the year 2021. Further research on negative emotions, such as panic and fear, and their effect on consumer behavior in other crisis contexts and economic contexts are suggested as future research to understand the influence of negative emotions on consumer purchasing decisions.
